# Observations on the Use of Instruments in Cases of Difficult and Protracted Labour

**Published:** 1831

**Authors:** 


### OBSERVATIONS ON THE USE OF INSTRUMENTS IN CASES OF DIFFICULT AND PROTRACTED LABOUR. By *John Beatty*, M.D. Licentiate of the King and Queen's College of Physicians in Ireland.

[Dublin Medical Transactions.]

THIS is so long and important a paper, that we think it right to dedicate an article to the analysis of it. There can be no doubt that, in 29 cases out of 30, Nature requires no assistance—at least instrumental assistance, in the process of human parturition. In such cases the accoucheur has only to wait and watch the occurrence of any unfavourable accident, or deviation from the ordinary course. Unfortunately a certain proportion of cases do occur, in which instrumental aid is necessary—and it is therefore our duty to inquire into the merits of the means proposed to assist delivery. It is often a difficult, and always an important task to discriminate between the necessity for instruments and the propriety of leaving Nature to her own course. After a long and active experience respecting the comparative value of the different instruments used in long-protracted labours, our author has formed his own conclusions, and has now been induced to lay a faithful account of his experience before the public. He acknowledges that his opinions on these important subjects, differ from those of some of his most eminent brethren in the obstetric line in his own city; but such difference of opinion must be expected to exist under all such circumstances. The cases in which mechanical assistance is required, may be comprised in two divisions.

"1st. Those where there is a disproportion between the head of the child, and the passage through which it must come; and 2dly. Those in which, although no mechanical impediment exists, the expulsive powers of the mother are not sufficient to accomplish the delivery.

Under the former will be found those caused by the deformity of the bony parietes of the pelvis, and by disease or rigidity of the soft parts, as well as unnatural size of the head of the fœtus, face presentations and transverse position of the head. And under the latter, those in which delivery is delayed by general weakness of the patient, hæmorrhage, frequent faintings, convulsions, great exhaustion, fever, &c.

To assist delivery under such circumstances, two classes of instruments have been devised; 1st. Those by which extraction may be effected without injury to either mother or child; 2dly. Those by which the life of the latter must necessarily be sacrificed. I need scarcely remind the members of an enlightened and humane profession, that the adoption of the latter alternative, is a step calling for the most serious consideration, and one that involves an awful, and heavy responsibility. The value of human life is not to be estimated by the age, nor is there in the eye of the law, either human or divine, any distinction between that of the octogenarian and the child unborn.

It matters little, therefore, what the nature of the situation is, in which a fellow-being committed to our care is placed, whether it be a fever striking him in the prime of life, or a disease requiring the performance of a capital operation, or the perils attending his first entrance into the world, it is our bounden duty to employ such means as will best insure his safety." 44.

By these observations Dr. Beatty would wish to be understood as overlooking, for a moment, the well-being of the mother, in the attempts to save the life of the child—he means that the life of the infant is to be sacrificed *only* when all means to preserve it, consistently with the safety of the mother, have been tried and proved inefficient. The perforator and crotchet were the instruments employed formerly—and, even so late as 1746, Lamotte states that, when surgeons were called in to cases of midwifery they took their instruments with them—" and brought away the child by their means." The circumstance of a woman being in labour a day and a half, or two days, was more than sufficient to set them to work—and this was their only resource in all cases indiscriminately. Fortunately a revolution, in this respect, was effected by Dr. Chamberlain, who, by the introduction of a harmless instrument, gave rise to an investigation into the true nature of difficult labours, and proved the utility and safety of the instrument. This physician's invention of the forceps brought about a classification of difficult labours, and led practitioners to discriminate between cases in which the life of the child must be sacrificed, and where it may be saved. At present, it is nearly the universal opinion that the perforator and crotchet should be the practitioner's last resource. The necessity for applying the forceps, and the period at which they should be applied, are not so easily settled. The urgency of a case cannot be estimated by the number of hours it has lasted, but by the state of the mother in each individual instance.

"Doctor Denman defines difficult labours to be, 'those in which, although the head of the child presents, the delivery is not terminated in twenty-four hours from the commencement of real labour.' Every practitioner must be aware that such cases are by no means unfrequent, and that the efforts of nature in very many of them, and even in others of much longer duration, are sufficient eventually to expel the child. But when labour is thus protracted, circumstances may, and do often render it desirable, to expedite delivery. These, as I have said, relate not to time, but to the condition of the mother; some women being able to bear a much greater length of suffering than others. In the more simple cases, those that are unconnected with convulsions, hæmorrhage, &c. the state of exhaustion of the mother, and cessation of labour-pains, are the best indications for the interference of art.

Doctor Osborne says, 'that in the state indicating the use of forceps, all the powers of life are exhausted, all capacity for further exertions is at an end, and the mind as much depressed as the body, they would both sink together under the influence of such continued and unavailing struggles.'

Now, to wait for such a period as this, is but to delay the operation, until the chances of success are almost lost; in fact there will be little prospect of any thing, but the removal of a dead child, from a dying mother; and it is such a practice, that has at times, brought this valuable instrument into disrepute and disuse: the want of success has been charged upon the operation, where it ought to be laid at the door of the operator. It is with us in this, as it is with the surgeon in strangulated hernia, the operation should be performed as soon as the necessity for it is found to exist, every moment's delay diminishing the prospect of a successful termination; and it is to this principle that so many happy results from the use of the scalpel in that disease, in modern times, are to be attributed. Let not the accoucheur, therefore, wait until the powers of life are exhausted; his duty is to prevent such an occurrence, and this is to be done by the timely application of the forceps. Delivery with this instrument may be attempted in whatever position the head may be, if it is sufficiently low in the pelvis, while at the same time the os uteri is dilated, or the soft parts are relaxed. As soon as matters are in this state, the practitioner should proceed to delivery without waiting until the mother's strength is so exhausted as to raise alarms for her safety, and oblige him to fly to any means of extraction, without regard to the life of the child. Delay under such circumstances, and running the patient to the last extremity, in giving her and nature (as it is called) every chance, is in my opinion, a main cause of the too frequent use of the perforator. Neque timerè, neque timidè, is the best motto by which the accoucheur can be guided in such circumstances." 50.

By this timely interference, he thinks, the evils attending upon difficult labours, such as contusions, inflammations, and sloughing of soft parts, would be obviated—and as to the bad effects said to follow the use of the forceps, he has never seen them, when the instruments were properly employed. In looking over his case-book, the author finds that, during 42 years, in which he had practised midwifery, five of which were spent in the Dublin Lying-in Hospital, he delivered 111 women with forceps or lever—and yet, in no instance of these 111 cases, did any unpleasant result follow. None of the mothers died—none suffered laceration of the perineum, nor any of those evils which are set forth as the effects of the forceps. All the children that were supposed to be living at the commencement of labour were born alive, and none had any injury or mark inflicted by the instrument.

"With respect to the operation, no great dexterity is required for its performance; a little management in the introduction of the blades, and patience in the extraction, is all that is required to bring it to a happy termination. The instrument I have always used, is that which is called male and female, from the transverse opening in the root of one blade, through which the other is passed—other practitioners prefer the curved forceps—it is quite immaterial which is chosen, provided they are used in proper time, and with good judgment.

Having ascertained by the rules already laid down, that immediate delivery is desirable, my custom is to empty the bladder and rectum, by the catheter or an enema if required. The patient being placed on her side, as near to the edge of the bed as possible, I proceed by introducing the female blade of the forceps, slowly and carefully over the upper side of the head of the child, until it reaches beyond the ear; this being accomplished, the chief difficulty is overcome, for the male blade being passed through the slit in the female blade, readily applies itself in the proper position, by gently urging it forward under the inferior side of the head. It is of importance to attend to this order of proceeding, for if the female blade were introduced to the under side, it would be difficult, from the relative position of the patient, and the bed, to pass the male blade through it. The application of the instrument usually brings on slight action of the uterus, although it may have ceased for several hours. This I always wait for, and taking advantage of the natural effort, the perineum being supported by the nurse-tender, or my own left hand, I have seldom found any difficulty in extracting the child alive and uninjured, provided it were so previous to the commencement of the operation. The operation as performed in this manner gives so little pain, and delivery is in general so easily accomplished by it, that I have been several times requested by patients, with whom I had previously employed forceps, to use them in subsequent labours." 53.

Our author has been called in to many cases where the perforator was ready on the table for the destruction of the fœtus, but where he brought it safe into the world by the forceps. It is no uncommon circumstance, he observes, to see children born alive and cry, whose heads had been opened, and the brains partially destroyed! Dr. Burns relates cases where children have lived in this mutilated state for a day or two. Mr. Dease states instances where "the child has been miserably dragged alive into the world, with a great part of the brain evacuated."

"Similar instances have (I understand) occurred in this city, in one of which humanity prompted the accoucheur to plunge the child into a vessel of water, to put an end to its existence and cries.

I can never forget a scene of horror to which I was a witness in the year 1800. I was called upon to see a very young lady, in labour of her first child, who was under the care of one of the oldest and most eminent practitioners in this city, (since dead;) her labour was most violent, which she bore with great impatience and noise. The head had been down on the perinæum (he said) several hours; I proposed to give more time, and an opiate, not doubting the powers of nature, or to try the forceps, which he declined, on account of its being her first child, and the apprehension he entertained of her being exhausted; and finally, he opened the head. The operation, as it always does, excited extraordinary uterine action, and before it was well concluded, or the brain evacuated, so as to lessen the bulk of the head, the child was propelled into the world alive and crying.

The old gentleman whose patient she was, was a person of very fine feelings, and the reader may imagine his sufferings on viewing the effect of a rash and ill-judged operation; he declared no earthly consideration should ever induce him again to witness the application of the perforator. 55

Some cases are next detailed, of which we shall abbreviate one or two.

*Case* 1. Mrs. M. aged 30 years, and very corpulent, took labour of her first child on the 28th November, early in the morning. At 6, p. m. the membranes burst; and from this date he commences the labour. The pains continued to increase in severity and frequency for a period of 68 hours, during the whole of which she had not slept, or taken any sustenance except a small quantity of whey. Her strength was considerably exhausted. The head was now sufficiently low in the pelvis—the os uteri dilated—and the external parts relaxed. The Doctor then applied the forceps, and waited for a pain, on the occurrence of which, he gave assistance, and a living boy was brought into the world without a scratch. This was a case, he thinks, where there was no likelihood of the labour being over for several hours, if left entirely to Nature, during which time there would have been considerable danger both to mother and child.

*Case* 2. Dr. B. was called to a patient who had been upwards of twenty-four hours in severe labour of her first child. The head was pretty low in the pelvis, though not on the perineum. The head of the child was much swollen, and Dr. B. had some difficulty in introducing the forceps, and then waited for a pain, three of which occurred and were assisted, without extraction. The fourth effort was successful, and a large living boy was born.

This was a case, Dr. B. observes, in which the head would certainly have been opened by those who are prejudiced against the forceps. The occurrence of convulsions, in cases of difficult parturition, has been considered as affording a sufficient ground for immediate delivery by perforation of the head. Without wishing to delay too long where the mother is in imminent danger, Dr. B. thinks that many lives might be saved, under such circumstances, by the forceps—especially as this last instrument does not require so long time as the perforator. The following case is given in illustration.

"In the year 1814, a gentleman, residing eighteen miles from Dublin, called on me, to request I would accompany him with all expedition to see his wife, who had been suddenly seized with labour of her first child, attended with convulsions before he left home. We reached his house in about five hours from the time he left it. I found the lady lying on the parlour-floor, labouring under severe convulsions, and quite insensible, in which state she had remained during her husband's absence. On examination, the head was found to be low in the pelvis, and the os uteri dilated. Without removing her I introduced the forceps, and in a few minutes succeeded in extracting a female child alive. The mother was now removed to bed; the convulsions ceased in a short time; her senses were restored, and the recovery was as speedy as if no untoward circumstance had occurred. I may observe that the gentleman had no more children, and the child then born is now alive, and heiress to his large estates; a consolation of which he must have been deprived, had I rashly employed a destructive instrument. If I had experienced much difficulty in this case, I would have thought myself justifiable, nay, called upon, to sacrifice the child, but certainly not until I knew it was unavoidable; and I state it to show that in the worst of cases, the milder means may be resorted to with considerable prospect of success." 59.

Dr. Beatty cautions us against drawing conclusions too precipitately from the real or estimated measurements of the head and pelvis, as reasons for craniotomy. It is not true that every female with a distorted spine has a deformed pelvis. We should endeavour to ascertain whether the deformity commenced before or after puberty. If the former, then there is reason to conclude that the pelvis has participated. If it did not come on till after the growth of the body was completed, we may hope to find no deformity in the pelvis. It is truly surprising to witness the degree of compression which the head of a child will bear without detriment. Dr. Denman relates the case of a child, born alive, with a depression full an inch in depth, on the left parietal bone, occasioned by a projection of the os sacrum. But the depressed part gradually rose, and, in a few months, regained its original level.

Dr. Beatty's paper is certainly deserving of the serious attention of the obstetric practitioner.

